# T Cell Metabolism: A New Perspective on Th17/Treg Cell Imbalance in Systemic Lupus Erythematosus

**DOI:** 10.3389/fimmu.2020.01027

**Published:** 2020-05-22

**Authors:** Juan Shan, Hong Jin, Yan Xu

**Affiliations:** Department of Immunology, School of Basic Medical Sciences, Chengdu Medical College, Chengdu, China

**Keywords:** cell metabolism, T helper 17 cells, regulatory T cells, systemic lupus erythematosus, mTOR signaling

## Abstract

The Th17/T-regulatory (Treg) cell imbalance is involved in the occurrence and development of organ inflammation in systemic lupus erythematosus (SLE). Metabolic pathways can regulate T cell differentiation and function, thus contributing to SLE inflammation. Increasingly, data have shown metabolism influences and reprograms the Th17/Treg cell balance, and the metabolic pattern of T cells is different in SLE. Notably, metabolic characteristics of SLE T cells, such as enhanced glycolysis, lipid synthesis, glutaminolysis, and highly activated mTOR, all favored Th17 differentiation and function, which underlie the Th17/Treg cell imbalance in SLE patients. Targeting metabolic pathways to reverse Th17/Treg imbalance offer a promising method for SLE therapy.

## Introduction

Systemic lupus erythematosus (SLE) is an autoimmune disease characterized by tissue inflammation and profound damage to multiple organs. The pathological mechanisms of SLE remain unclear; however, it has been reported that the imbalance between T helper 17 (Th17) and regulatory T cells (Tregs) underlies the pathogenesis of SLE (1–4). Th17 cells have pro-inflammatory effects, and the proportion of Th17 cells is higher in SLE patients, and the content is positively correlated with the activity of SLE disease (5). Tregs, however, have immunosuppressive function and play an important role in the induction and maintenance of self-tolerance. The reduced content and dysfunction of Tregs are closely related to the occurrence and development of SLE (6–8). Tregs injected into lupus mice can control the inflammatory response and alleviate pathological damage (9). Thus, improving the Th17/Treg cells imbalance shows some promise for the treatment of SLE.

Th17 and Treg cells are mutually antagonistic in function and differentiation. Many mechanisms regulating Th17/Treg cells balance have been reported. Besides the molecular signaling network, accumulating evidence has shown that cellular metabolism is also critically involved in Th17 and Treg differentiation (10). These two cell subsets are dictated by distinct metabolic pathways, and manipulating metabolic pathways can regulate Th17/Treg cells balance. In T-cells from SLE patients and lupus-prone mice, metabolic abnormalities linked to Th17/Treg cell imbalances have been reported. Here, we reviewed how cellular metabolism influences Th17 and Treg cell differentiation, summarized metabolic abnormalities of SLE T cells, and we here propose that metabolic abnormalities of SLE T cells is the mechanism by which the Th17/Treg imbalance emerges in SLE patients. Manipulating cellular metabolism to correct aberrant immune responses may be a suitable means of treating SLE.

## Metabolic Control of Th17/Treg Balance

T-cell metabolism is highly dynamic. The metabolic pattern changes during the process of activation, proliferation, and differentiation. Naive T cells have low energy requirements. They import a small amount of glucose and generate ATP mainly through the TCA cycle and OXPHOS. Upon activation, T cells start to proliferate and differentiate. They need to reprogram their metabolic pattern to meet their bioenergetic and biosynthetic requirements. A variety of metabolic substrates (glucose, amino acids, and fatty acids) and metabolic pathways (glycolysis, oxidative phosphorylation, the pentose phosphate pathway, fatty acid synthesis and oxidation, and glutamine metabolism) are mobilized to adapt to their biological functions. Glycolysis can rapidly produce ATP to provide the energy needed to rapidly proliferate, and a large number of intermediate products of various metabolic pathways allow biosynthesis (11–14).

Studies have shown that T helper cell subsets have different metabolic patterns, of which those of Th17 cells and Tregs are the most distinct. One study evaluated 400 energy metabolites, metabolism-related genes, and proteins in Th17 and Tregs and results indicated that Th17 cells contain high levels of pyruvate, lactic acid, early glycolysis, and pentose phosphate pathway intermediates, and they express key proteins in glycolysis pathway, like Glut-1 and HK-2, at high levels. Tregs had higher C2 and C4-OH carnitine levels and more expression of fatty acid transporter CPT1A and electron transport chain component cytochrome c, which suggested that Th17 cells were mainly powered by glycolysis, and the pentose phosphate pathway was also active; Tregs, on the other hand, were found to rely more on fatty acid oxidation and oxidative phosphorylation to supply energy (15). Glycolysis deprivation was found to impair Th17 differentiation dramatically, while defective glycolysis supported the development of Treg cells. Replacement of glucose with galactose, treatment with 2-DG (an inhibitor of hexokinase, the first rate-limiting enzyme of glycolysis), and lack of HIF1α, Cdc42, ICER, and mTOR (crucial regulators of T cell glycolytic metabolism) all resulted in diminished Th17 development but enhanced Treg cell differentiation (16–20). Conversely, inhibition of fatty acid oxidation results in diminished differentiation to Th17 cells, but increased development of Tregs (21).

Glutaminolysis is also preferentially increased in Th17 cells. TCA-cycle intermediates produced after α-ketoglutarate (α-KG) in Th17 cells were more plentiful than in Tregs. α-kG is also a metabolite of glutamine (15), suggesting increased glutaminolysis in Th17 cells. ICER, a transcriptional factor that enhances glutaminase1 and promotes glutaminolysis, is also expressed in large quantities in Th17 cells (22). In addition, glutamine metabolite 2-hydroxyglutarate could hypermethylate Foxp3 gene locus and inhibit Foxp3 transcription, thus promoting the differentiation of Th17 cells, which regulates Th17/Treg balance by an epigenetic mechanism (23).

Th17 cells have considerable fatty acid synthesis activity. The expression of ACC1, a key enzyme in fatty acid synthesis, was found to be significantly higher in Th17 cells than in Tregs. Drug inhibition or T-cell specific knockout of ACC1 could inhibit Th17 differentiation and promote the induction of Tregs both *in vivo* and *in vitro* (24, 25). Cholesterol intake and synthesis were also significantly higher in Th17 cells (26), leading to the accumulation of the cholesterol precursor, desmosterol, which acts as a potent endogenous RORγ agonist and dictates Th17 differentiation (27). Statins, a class of drugs that inhibit cholesterol biosynthesis, are reported to target Th17/Treg imbalance and alleviate Th17-mediated inflammatory response (28). The distinct metabolic patterns of Th17 and Treg cells provide a basis for intervention of Th17/Treg imbalance.

## Metabolic Abnormalities in SLE T Cells

Cell metabolism regulates the differentiation and function of T cells, thereby participating in the occurrence and development of SLE inflammation. Metabolic abnormalities in T cells from SLE patients and lupus-prone mice were reported (29, 30). These are characterized by the following:

(**1) Mitochondrial dysfunction:** T cells from SLE patients showed elevated mitochondrial transmembrane potential, increased ROS production and reduced ATP synthesis (31).

(**2) Hyperactivated glucose metabolism:** CD4^+^ T cells from SLE patients and lupus-prone mice have higher OCR and ECAR levels (32, 33), suggesting they have elevated levels of both glycolysis and oxidative phosphorylation. The metabolites of pentose phosphate pathway, such as R5P and F6P, were also higher in peripheral blood lymphocytes of SLE patients (34). These results suggested that three main pathways of glucose metabolism—aerobic glycolysis, pentose phosphate pathway, and oxidative phosphorylation—are involved in T cell activation in SLE patients.

(**3) Lipid synthesis enhancement:** there was more synthesis of lipid rafts in CD4^+^T cells of SLE patients than in normal controls, and inhibiting the synthesis of lipid rafts could alleviate the pathological manifestations of lupus in mice (35–37). Glycosphingolipids and cholesterol are important components of lipid rafts, and the levels of synthesis were significantly higher in CD4+T cells in SLE patients than in normal controls (38).

**(4) Increased glutaminolysis:** There is more expression of ICER, the transcriptional factor that promotes glutaminolysis and Th17 generation in CD4^+^T cells from SLE patients than in healthy controls (39). Glutaminase 1 inhibition improved autoimmune pathology in MRL/lpr mice, and suppressed Th17 differentiation of T cells from patients with SLE but not in those from healthy donors (40). Those data suggested increased glutaminolysis in SLE T cells.

**(5) Highly activated mTOR:** mechanistic target of rapamycin (mTOR) is a hub in the cellular metabolic signal network, regulating cellular growth and energy utilization. CD4^+^ T cells from SLE patients and lupus-prone mice showed increased mTOR activation (32, 33, 41, 42). The hyperpolarization of mitochondria and the overactivity of pentose phosphate pathway led to enhanced mTOR activity (43). In turn, highly activated mTOR can enhance glycolysis and fatty acid synthesis, thus promoting Th17 differentiation (44, 45). It can be seen that high mTOR expression is an important signaling mechanism leading to abnormal T cell metabolism and Th17/Treg imbalance in SLE patients. Many clinical trials in 2018 showed that sirolimus, an mTOR inhibitor, could alleviate the disease activity of SLE patients (46–48), expand their Foxp3^+^ Treg cells, and inhibit the secretion of cytokines such as IL-17 (46).

## Dicussion

**Figure 1:**
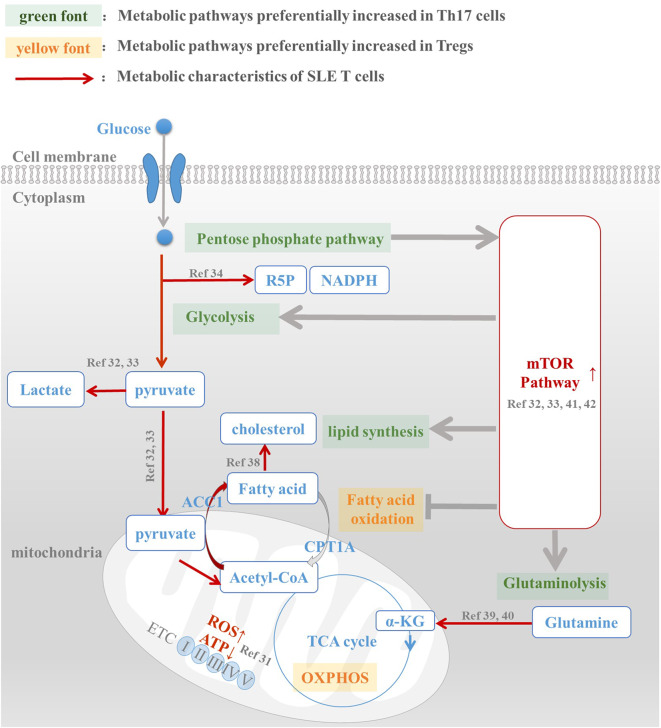
Metabolic abnormalities is the underlying mechanism of Th17/Treg imbalance in SLE patients. Th17 and Treg cells have a distinct metabolic pattern: pentose phosphate pathway, glycolysis, fatty acid synthesis, and glutaminolysis are preferentially increased in Th17 cells, while fatty acid oxidation and oxidative phosphorylation are active in Treg cells. However, metabolic abnormalities of T cells in SLE patients, including enhanced glycolysis, active lipid synthesis, increased glutaminolysis, and high mTOR activation, are all conducive to Th17 differentiation and function. For this reason, we speculated that abnormal T cell metabolism was the mechanism underlying Th17/Treg imbalance in SLE patients. The intervention of metabolic pathways to reprogram T cell metabolic patterns in SLE patients, reduce their overactivated glycolysis and lipid synthesis levels, and promote the oxidation of fatty acids, is expected to reverse the Treg/Th17 imbalance in patients and restore their normal immune function.

The abovementioned metabolic characteristics of SLE T cells, such as enhanced glycolysis, lipid synthesis, glutaminolysis, and highly activated mTOR, all favored Th17 differentiation and function, which suggest the metabolic abnormalities of SLE T cells is the underlying mechanism of Th17/Treg imbalance in SLE patients (Figure 1). Inhibition of glycolysis (32, 33), lipid synthesis (28, 36–38), and mTOR signaling (46–48) can control inflammation and alleviate disease activity in lupus mouse and SLE patients. Manipulating cellular metabolism to correct aberrant immune responses offers promising method for SLE therapy. Further studies are still needed to explore the metabolic abnormalities occurring in T cells of SLE patients and their role in disease progression, as well as how they response to therapies, especially those have potential role in intervening Th17/Treg cell imbalance.

In addition to drugs targeting metabolic pathways, dietary habits, and nutritional factors can also modulate Th17/Treg balance by affecting T cell metabolism. Low cholesterol diet could improve Th17/Treg balance by the activation of LXRs (49), nuclear receptors that modulate cholesterol metabolism (50). And high glucose intake was found to exacerbate autoimmunity by inducing Th17 cells via upregulation of mitochondrial ROS in T cells (51). The long-chain fatty acids enhanced differentiation of Th17 cells, while the short-chain fatty acids derived from a fiber-rich diet expanded Treg cells and reduce IL-17 production (52–54). Thus, a balanced diet could be helpful in the prevention and management of SLE (55).

## Author Contributions

JS, HJ, and YX wrote the manuscript. JS generated themes and ideas, guided, and edited the manuscript.

## Conflict of Interest

The authors declare that the research was conducted in the absence of any commercial or financial relationships that could be construed as a potential conflict of interest.
